# Clinical Outcomes of Extracorporeal Membrane Oxygenation for Acute Respiratory Distress Syndrome in Patients with Traumatic Brain Injury or Spontaneous Intracerebral and Subarachnoid Hemorrhages: A Retrospective PILOT Study

**DOI:** 10.3390/medicina62010013

**Published:** 2025-12-21

**Authors:** Minkeun Song, Solji Jung, Kyeong-O Go, Kwangho Lee, Won Heo, Soo-Hyun Hwang, Hyun Park, Joung Hun Byun, Dong Hoon Kang, Jong Woo Kim, Sungdae Lim

**Affiliations:** 1 Department of Neurosurgery, Gyeongsang National University Changwon Hospital, Gyeongsang National University School of Medicine, 11 Samjeongja-ro, Seongsan-gu, Changwon 51472, Republic of Korea; santahouse9@naver.com (M.S.); 65429@naver.com (S.J.); kko8353@gmail.com (K.-O.G.); serapis79@naver.com (K.L.); wonni8@naver.com (W.H.); mdshhwang@naver.com (S.-H.H.); 2 Department of Neurosurgery, Gyeongsang National University Hospital, Gyeongsang National University School of Medicine, 79 Gangnam-ro, Jinju 52727, Republic of Korea; 1coo3004@naver.com; 3 Department of Thoracic and Cardiovascular Surgery, Gyeongsang National University Changwon Hospital, Gyeongsang National University College of Medicine, 11 Samjeongja-ro, Seongsan-gu, Changwon 51472, Republic of Korea; jhunikr@naver.com (J.H.B.); drk82@hanmail.net (D.H.K.); cs99kjw@hanmail.net (J.W.K.)

**Keywords:** extracorporeal membrane oxygenation, respiratory distress syndrome, brain injuries, traumatic, cerebral hemorrhage, subarachnoid hemorrhage, intensive care units

## Abstract

*Background and Objectives*: The usefulness of extracorporeal membrane oxygenation (ECMO) therapy for acute respiratory distress syndrome (ARDS) in patients with traumatic brain injury (TBI), spontaneous intracerebral hemorrhage (ICH), or subarachnoid hemorrhage (SAH) is limited. This study aimed to investigate the survival benefit of ECMO in ARDS that developed during hospitalization in high-risk neurological patients. *Materials and Methods*: This retrospective study evaluated patients with TBI or spontaneous ICH and SAH admitted to our hospital’s neurosurgery intensive care unit and who received ECMO therapy for ARDS between March 2017 and March 2024. Clinicodemographic characteristics, indications for and methods of ECMO application, occurrence of comorbidities, hospital survival rates, and ECMO weaning success rates were compared between survivors and non-survivors. *Results*: Among the 16 patients evaluated, seven (43.8%) were successfully discharged. The mean ages of the survivor and non-survivor groups were 52.1 and 58.6 years, respectively. The PaO_2_/FiO_2_ ratio pre-ECMO was similar in both groups (66.6 vs. 69.2); however, it improved significantly post-ECMO in the survivor group (264.1 vs. 209.4). The ECMO success rate was 100% in the survivor group and only 33.3% in the non-survivor group. The intensive care unit and hospital lengths of stay were longer in the survivor group. Most patients received veno-venous ECMO, and hemorrhagic complications were rare. *Conclusions*: ECMO for ARDS in patients with severe TBI or spontaneous ICH and SAH positively impacts survival and functional recovery and may be a beneficial treatment modality.

## 1. Introduction

Traumatic brain injury (TBI), spontaneous intracerebral hemorrhage (ICH), and subarachnoid hemorrhage (SAH) are severe neurological diseases frequently encountered in neurosurgical intensive care units. In addition to early disease changes, secondary prognostic deterioration due to multiple organ failure and pulmonary complications is common. In particular, acute respiratory distress syndrome (ARDS) developing from complications (e.g., pneumonia during mechanical ventilation), aspiration, or the use of sedative drugs markedly increases the mortality rate. ARDS renders conventional respiratory treatment alone insufficient for maintaining oxygenation [1]. ARDS occurs in approximately 20–30% of patients with severe brain injury and is a major influencing factor of worse prognosis, prolonging mechanical ventilation, increasing intracranial pressure, and increasing mortality rates [2]. Extracorporeal membrane oxygenation (ECMO) has been recently reported to be beneficial for various indications, such as severe infections, trauma, and cardiac arrest. The use of ECMO as salvage therapy for severe ARDS has also gradually expanded [3,4]. The recovery pattern after ECMO highly influences prognosis [5].

The application of ECMO in patients at risk of intracranial hemorrhage remains limited owing to concerns regarding anticoagulant use and the possibility of bleeding recurrence [4]. However, increasing evidence supports that ECMO can improve survival rates in patients with persistent hypoxemia from respiratory failure, in whom ensuring adequate oxygenation to prevent the progression of brain injury is the main priority [4,5]. This study aimed to investigate the survival benefit of ECMO in high-risk neurological patients. Accordingly, we analyzed the clinical outcomes of patients with TBI or with spontaneous ICH and SAH who received ECMO therapy for ARDS that developed during hospitalization.

## 2. Materials and Methods

This single-center retrospective study was approved by the appropriate institutional review board (IRB No. 2025-006-003) and was conducted according to the tenets of the Declaration of Helsinki. The need for informed consent was waived owing to the retrospective observational nature of the study [6].

Patients with TBI or ICH admitted to our hospital’s neurosurgery intensive care unit between March 2017 and March 2024 were evaluated for eligibility. The inclusion criterion was receiving ECMO therapy for ARDS that developed during respiratory treatment. Patients were identified based on electronic medical records, and data from imaging studies, patient records, and intensive care logs were collected. The following data were collected: age, sex, underlying disease, primary brain lesion, initial Glasgow Coma Scale (GCS) score, time of ARDS onset, use of anticoagulants, method and initiation time of ECMO, ECMO maintenance duration, duration of artificial respiratory treatment, occurrence of hemorrhagic complications, and in-hospital mortality. Considering that the purpose of ECMO extends beyond oxygenation support to spontaneous functional recovery, weaning success may serve as an important indicator of survival [5]. Hence, the rate of ECMO weaning success was also analyzed.

Survival was classified and recorded as intensive care unit (ICU) and hospital survival, and long-term prognosis was evaluated based on modified Rankin scale (mRS) scores at discharge and at 3- and 6-month follow-up. ARDS was diagnosed as PaO_2_/FiO_2_ ratio <150, persistent bilateral pulmonary infiltration, and mechanical ventilation dependency according to the Berlin definition [4]. ECMO was considered when the oxygenation index (PaO_2_/FiO_2_ ratio, a key indicator of treatment response to ECMO in patients with severe ARDS [5]) was consistently <100 or for severe respiratory failure unresponsive to conventional therapy. The ECMO type (veno-venous [VV] or veno-arterial-venous [VAV]) was selected through a consultation between the cardiovascular surgery specialist and the attending neurosurgeon [7].

Comparative analyses were performed between the survivor and non-survivor groups. Continuous variables are presented as mean ± standard deviation and were compared using Student’s t-test or the Mann–Whitney U test based on the normality of data distribution. Categorical variables were expressed as frequencies and percentages, and differences between the two groups were analyzed using Fisher’s exact test or the χ^2^ test. Disease severity and prognosis were compared according to the timing of ECMO administration (early [within 5 days of ARDS onset] vs. [5 days after ARDS onset]). All statistical analyses were performed using Python (version 3.9)-based Pandas and SciPy packages. Statistical significance was defined as a two-sided *p*-value of <0.05.

Given the retrospective design, analyses were conducted using the available complete data. Patients with missing values for key clinical variables were excluded from the comparisons.

## 3. Results

Sixteen patients were evaluated. The mean patient age was 55.9 years (range, 27–82 years), and 11 (68.8%) patients were men. Overall, 3 (18.8%) and 13 (81.2%) patients had TBI and spontaneous ICH and SAH, respectively. The characteristics of the patients are described in Table 1.

A total of seven (43.8%) patients survived, while nine (56.2%) patients died. Among the seven survivors, five showed a favorable neurological outcome, with an mRS score of 3 or lower at the time of discharge. At the 3-month follow-up, three of these patients maintained or improved their functional status, and by 6 months, two had achieved near-complete recovery with an mRS score of 1. One patient was lost to follow-up after discharge, and long-term outcome data were unavailable for that patient. The comparison of patient characteristics between the survivor and non-survivor group is shown in Table 2. The mean age was different between the survivor and non-survivor groups, but the difference was not significant (52.1 ± 19.0 vs. 58.6 ± 15.8 years, *p* = 0.486).

**Figure 1:**
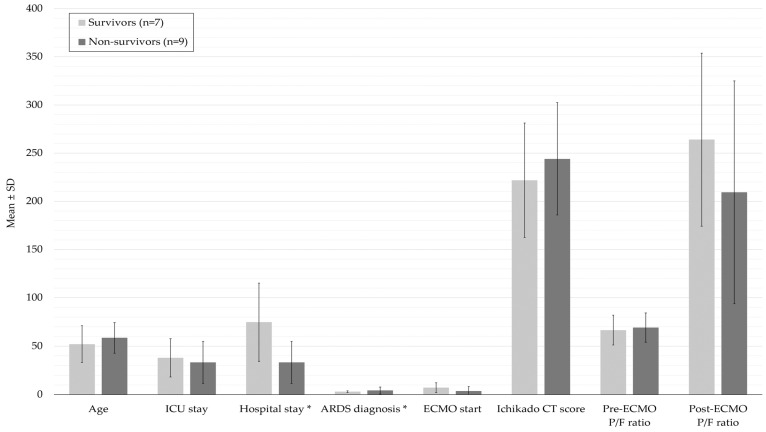
Comparison of clinical variables and severity scores between the survivor group and the non-survivor group. Clinical variables include age, ICU length of stay, hospital length of stay, the timing of ARDS diagnosis and the timing of ECMO initiation since ARDS diagnosis. Severity scores include Ichikado CT score evaluating radiologic severity and P/F ratio evaluating physiological severity. Each bar represents the mean value with standard deviation (± SD) for each group. Variables with statistical significance are marked with an asterisk (*).

Table 2 summarizes analysis of general considerations when applying ECMO between the survivor group and the non-survivor group. Although the pre-ECMO PaO_2_/FiO_2_ ratio evaluating the physiological severity of ARDS was not significantly different between the survivor and non-survivor groups (66.6 ± 15.4 vs. 69.2 ± 15.1, *p* = 0.74), the PaO_2_/FiO_2_ ratio post-ECMO was higher in the survivor group (264.1 ± 89.8 vs. 209.4 ± 115.6) (Figure 1). The Ichikado CT scoring evaluating the radiological severity of ARDS was not significantly different between the two groups. There was no significant difference in the ICU length of stay between the survivor and non-survivor groups (38.0 ± 19.7 days vs. 33.3 ± 21.6 days, *p* = 0.964). However, the hospital length of stay was significantly longer in the survivor group (74.9 ± 40.4 days vs. 33.3 ± 21.6 days, *p* = 0.042) (Table 2). The ECMO weaning success rate was also significantly higher in the survivor group than in the non-survivor group (100% (7/7) vs. 33.3% (3/9), *p* = 0.011) (Table 2). The mRS score recovered to ≤3 at discharge in five patients, and the two of them had an mRS score of 1 at the 6-month follow-up.

In the comparison according to the timing of ECMO initiation, the GCS scores were not significantly different between the early and delayed groups (10.5 vs. 9.5, *p* = 0.64). Meanwhile, the delayed ECMO group had a longer ECMO duration (26.8 vs. 11.3 days) and ICU and hospital lengths of stay. However, the hospital survival rate was higher in the delayed group (75.0%) than that in the early group (33.3%). The mRS score at discharge was also higher in the delayed group (3.5 vs. 4.6), although the difference was not significant (*p* > 0.05, Table 3).

## 4. Discussion

### 4.1. Main Findings

This study shows that ECMO treatment is not only beneficial for survival but also for better long-term functional prognosis in patients with severe TBI or spontaneous ICH and SAH. Among the 16 patients who received ECMO in the current study, seven (43.8%) survived, and 6 had an mRS score of ≤3 at discharge. Furthermore, 5 of these seven patients achieved independent functional recovery, with an mRS score of 1 at the 6-month follow-up.

### 4.2. ECMO for ARDS

ARDS in patients with TBI or ICH is a major comorbidity that makes treatment difficult, and current conventional treatment strategies have limited survival benefits. This is primarily because patients with TBI or SAH cannot be placed in the prone position owing to the risk of increased intracranial pressure. Further, the use of sedatives and neuromuscular blockers can interfere with neurological assessments. If refractory hypoxemia is not controlled despite conventional mechanical ventilation and lung-protective strategies, ECMO is a reasonable treatment option. However, the application of ECMO is limited in patients with hemorrhagic cerebrovascular disease owing to the risk of exacerbated bleeding from anticoagulation therapy [8].

The survival rate in the current study was higher than the low survival rate previously reported in similar populations managed without ECMO. Although Elmer et al. found ICU mortality approaching or exceeding 70% in patients with spontaneous ICH complicated by ARDS, highlighting the exceptionally poor prognosis in hemorrhagic cerebrovascular disease without ECMO support [9]. Additionally, a meta-analysis of 16 TBI ECMO studies (*n* = 383) reported a pooled survival rate of only 66.1%, suggesting that conventional therapy alone confers a limited survival benefit [10]. Similarly, Shen et al. reported an ICU mortality rate exceeding 70% in patients with ICH who developed ARDS, indicating a significantly poorer prognosis than in general ARDS patients [11]. The hospital survival rate of the ECMO-treated patients in the current study was more than two times higher than that in these previous reports. In particular, the mean change in the PaO_2_/FiO_2_ ratio (ΔP/F) before and after ECMO was higher in the survivor group than in the non-survivor group (197.5 vs. 140.2), suggesting that the physiological response to ECMO may be related to survival. Collectively, these results indicate that ECMO can be considered not only for survival but also for prognostic improvement in patients with brain injury. ECMO can be an effective respiratory support strategy in neurosurgery intensive care units, where it is difficult to place patients in the prone position or apply intensive mechanical ventilation.

Similar trends have been reported in previous studies. Combes et al. emphasized that although ECMO could improve survival rates in patients with severe ARDS, patient selection was more important than the timing of application [8]. Schmidt et al. reported that the PRESERVE score, reflecting pre-ECMO survival indicators, was useful for prognosis prediction [12]. Accordingly, recent Extracorporeal Life Support Organization (ELSO) guidelines recommend application strategies based on biological indicators rather than on simple time criteria [13]. The results of this study are consistent with this recommendation. However, this was a retrospective single-center study, and the decision to apply ECMO was made based on clinical judgement without clear criteria; therefore, a selection bias may exist. Nevertheless, the results suggest that ECMO can help improve survival, even in patients undergoing neurosurgery. The findings can be used as a basis for future multicenter comparative studies and for developing ECMO application guidelines based on individual patient risks.

Extracorporeal membrane oxygenation (ECMO) improves survival in patients with severe ARDS. In a large meta-analysis of individual patient data, Combes et al. demonstrated that ECMO significantly reduced 90-day mortality compared to conventional mechanical ventilation (36% vs. 48%; relative risk 0.75, *p* = 0.013) [8].

In patients with hemorrhagic brain injuries, the use of ECMO raises concerns regarding the risk of rebleeding due to systemic anticoagulation. However, recent evidence suggests that ECMO may still be a viable option in selected cases. Luyt et al. reported that, among trauma patients-including those with TBI or intracranial hemorrhage-venovenous ECMO was not commonly associated with worsening intracranial bleeding [14]. Moreover, when anticoagulation was carefully managed and neurological monitoring was performed, many survivors achieved favorable neurological outcomes.

In the current study, only one patient developed severe hemorrhagic complications directly related to ECMO, and treatment continued with aggressive intracranial pressure control and an anticoagulation strategy. This shows that systemic anticoagulant control and intracranial pressure monitoring can help minimize major hemorrhagic complications, highlighting the importance of a multidisciplinary strategy when performing ECMO [15].

#### 4.2.1. Relationship of ECMO Timing with Disease Severity and Prognosis

Previous studies have suggested that early ECMO can improve prognosis by improving lung protection and oxygenation. However, the current study found no significant difference in prognosis between patients who underwent early (within 5 days after ARDS onset) and delayed (5 days after onset) ECMO, with no significant difference in GCS scores between these patients. With respect to treatment characteristics, despite the longer ECMO duration and ICU and hospital lengths of stay in the delayed group, the group had a higher hospital survival rate than the early group (75.0% vs. 33.3%). Furthermore, the mRS score also showed a more favorable trend in the delayed group (3.5 vs. 4.6), although the difference was not significant (*p* > 0.05, Table 3). These results suggest that the early ECMO group may have included patients with worse disease severity and that, importantly, evaluating the timing of ECMO initiation based only on time criteria has limitations. In neurocritical care, comprehensive factors such as intracranial pressure, risk of hemorrhage, and anticoagulation therapy influence the decision for ECMO. Therefore, more accurate guidelines are required.

#### 4.2.2. Considerations Regarding the Timing of ECMO Application

The optimal timing of ECMO in patients with ARDS remains controversial. In general, ECMO is considered when adequate oxygenation cannot be maintained using conventional mechanical ventilation strategies or when there is a risk that ventilator management may cause further lung injury. However, very early ECMO may increase the risk of unnecessary invasive treatment, whereas very late ECMO may have limited effectiveness as multiorgan failure has already progressed. Combes et al. reported that although ECMO could improve survival rates in patients with severe ARDS, patient selection was more important than the timing of application [8]. Schmidt et al. highlighted that the PRESERVE score, reflecting physiological status pre-ECMO, is useful for predicting prognosis [12]. The results of the current study are consistent with previous findings that appropriate patient selection and severity assessment may be more important influencing factors of prognosis than ECMO application itself.

**Figure 2:**
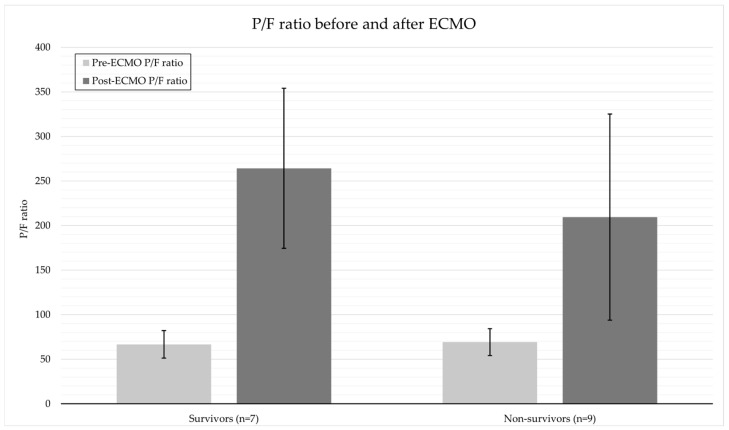
Comparison of PaO_2_/FiO_2_ ratio before and after ECMO support between the survivor and non-survivor groups. Although the post-ECMO PaO_2_/FiO_2_ ratio is higher in the survivor group, the difference is not statistically significant (*p* = 0.305). This trend may reflect a relatively greater recovery of oxygenation following ECMO among survivors. Bars indicate mean values with standard deviation.

When we compared the patients who underwent ECMO in the early (median 1.6 days) or late (medial 9.5 days) period after VAP diagnosis, the early ECMO group had a lower survival rate (12.5%). These patients had more severe conditions, as indicated by their lower GCS scores and PaO_2_/FiO_2_ ratios. These findings indicate that patient severity exerts a greater impact than timing of the decision to apply ECMO (Figure 2). Recent guidelines recommend determining the appropriate timing of ECMO application not only based on time criteria but also on the following physiological and clinical indicators: PaO_2_/FiO_2_ ratio <80 mmHg sustained for more than 6 h; driving pressure >15 cmH_2_O; Murray score ≥3; pH <7.20 with PaCO_2_ >80 mmHg (refractory hypercapnia); and failure of conventional treatment (e.g., prone position, neuromuscular blocking agents) [13,16,17]. The EOLIA trial showed that a PaO_2_/FiO_2_ ratio <50 for >3 h or <80 for >6 h could be an indication for ECMO, particularly its early application. In the current study, neither the physiological P/F ratio nor the radiologic severity score showed a significant difference between the survivor group and the non-survivor group when considered alone. Also, the timing of ECMO based on clinical judgement may have influenced the results because the severity assessment criteria for the early ECMO group were not standardized. Therefore, ECMO application algorithms based on severity scoring systems (e.g., PRESERVE and RESP) need to be established.

### 4.3. Limitations

This study has limitations. First, there are inherent limitations in the generalizability and heterogeneity within the patient group owing to the single-center, small-scale, and retrospective nature of the study. Second, there was no comparison group (e.g., patients with TBI/ICH+ARDS who did not receive ECMO), making it difficult to directly evaluate the effectiveness of ECMO. However, the 43.8% survival rate in the ECMO group in this study is markedly higher than the previously reported survival rate of 16–30% with conventional therapy for severe ARDS in patients with TBI or ICH [14,15]. Third, some patients were lost during long-term follow-up; therefore, the 6-month mRS analysis was incomplete. Despite these limitations, the importance of this study lies in its data showing the benefit of ECMO in improving survival and functional recovery in high-risk neurosurgery patients. Future studies should establish standardized protocols regarding the indications for ECMO use, anticoagulant strategies, and monitoring techniques (e.g., intracranial pressure, continuous electroencephalography, and serial computed tomography). The efficacy and safety of ECMO in these patients also need to be clarified more definitively through prospective multicenter studies that include a control group.

Furthermore, no statistical imputation was performed for missing data. All analyses were based on the available complete-case records, which may have introduced some selection bias.

## 5. Conclusions

ECMO, when applied through careful patient selection and a multidisciplinary approach, may help improve survival and functional prognosis in patients with TBI, spontaneous ICH, and SAH who develop ARDS. Particularly, for patients with severe ARDS who do not achieve adequate oxygenation despite mechanical ventilation, ECMO positively impacts survival, demonstrating the importance of an active respiratory support strategy even in patients with brain lesions.

## Figures and Tables

**Table 1:** Patient characteristics (*n* = 16). Abbreviations: BG, basal ganglia; DAI, diffuse axonal injury; EDH, epidural hematoma; ICH, intracerebral hemorrhage; IVH, intraventricular hemorrhage; AcomA, anterior communicating artery; MCAB, middle cerebral artery bifurcation; SCA, superior cerebellar artery; an., aneurysm; MMD, Moyamoya disease; SAH, subarachnoid hemorrhage; SDH, subdural hematoma; VA, venoarterial; VAV, veno-arterial-venous; VV, veno-venous.

Patient ID	Age (Years)	Sex	Diagnosis	GCS Score at Admission	ECMO Type	ECMO Duration (Days)	Anticoagulant (Heparin) Use (Y/N)	Bleeding Complications (Y/N)	Pre-ECMO PaO_2_/FiO_2_ Ratio	Post-ECMO PaO_2_/FiO_2_ Ratio	ECMO Weaning Success (Y/N)	Hospital Survival (Y/N)
1	77	F	Lt. BG, thalamic ICH c IVH	E2V1M4	VA	3	N	N	76.4	187	N	N
2	48	M	IVH	E2V4M5	VV	2	5000 U × 2 days	N	54	317.5	Y	Y
3	61	F	Pontine hemorrhage c IVH	E3V4M6	VAV then VA	7	5000 U × 7 days	N	74.4	158	Y	Y
4	75	F	Rt. Frontal SDH, SAH	E4V4M6	VV	14	10,000 U × 2 days	N	77.1	135	N	N
5	68	F	SAH d/t AcomA an. Ruptured	E1V1M2	VVA	2	25,000 U	N	49	157.5	N	N
6	84	F	Lt. frontal EDH	E2V3M6	VV	9	21,000 U	N	67.8	288	Y	Y
7	59	M	SAH d/t Lt. MCAB ab. Ruptured	E1V1M3	VV	32	8000 U, 6000 U, 1200 U × 5 days, 6000 U × 10 days	N	72	147.7	N	N
8	33	M	Rt. BG ICH c IVH	E2V2M5	VV-VV	36	25,000 U × 3 days	N	47	407.8	Y	Y
9	44	M	Lt. BG ICH c IVH	E3V4M6	VV	12	5000 U × 2 days	N	94.3	277.5	N	N
10	56	M	SAH d/t AcomA an. Ruptured	E3V5M6	VV	18	1000 U	Y	70	166	Y	N
11	64	M	Rt. Parietal ICH c IVH	E4V2M6	VAV then VV	20	N	N	74	490	Y	N
12	66	M	Cerebellar ICH	E3V4M6	VV	30	N	Y	86	248	Y	Y
13	39	M	Rt. Putaminal ICH c IVH, MMD	E3V5M6	VV	8	N	N	54	152	Y	Y
14	26	M	SAH d/t SCA an. ruptured	E3V1M3	VV	18	2500 U	N	44	113.8	Y	N
15	34	M	Lt. frontal contusional hemorrhage, DAI	E4V1M5	VV	15	N	N	83.3	277.5	Y	Y
16	58	F	Cerebellar ICH	E4V4M6	VV	16	5000 U × 3 days	N	66	210	N	N

**Table 2:** Comparison of patient characteristics between survivors and non-survivors. Values are presented as the mean ± standard deviation unless otherwise indicated. *p*-values are calculated using the Student’s t-test for continuous variables and Fisher’s exact test for categorical variables. * *p* < 0.05, statistically significant. Abbreviations: CT, computed tomography, ARDS, acute respiratory distress syndrome; ECMO, extracorporeal membrane oxygenation; ICU, intensive care unit.

Variable	Survivors (*n* = 7)	Non-Survivors (*n* = 9)	*p*-Value
Age (years)	52.1 ± 19.0	58.6 ± 15.8	0.486
Ichikado CT scoring	221.9 ± 59.4	244.1 ± 58.3	0.469
Pre-ECMO PaO_2_/FiO_2_ ratio	66.6 ± 15.4	69.2 ± 15.1	0.745
Post-ECMO PaO_2_/FiO_2_ ratio	264.1 ± 89.8	209.4 ± 115.6	0.305
ICU length of stay (days)	38.0 ± 19.7	33.3 ± 21.6	0.694
Hospital length of stay (days)	74.9 ± 40.4	33.3 ± 21.6	0.042 *
ARDS diagnosis day (HOD)	3.2 ± 0.8	4.3 ± 3.6	0.032 *
ECMO start day (from ARDS diagnosis)	7.2 ± 5.1	3.8 ± 4.5	0.651
ECMO weaning	7/7	3/9	0.011 *

**Table 3:** Comparison of patient characteristics and outcomes according to timing of ECMO initiation. Values are presented as mean ± standard deviation. Early ECMO group includes initiation within 5 days of ARDS onset, delayed ECMO group includes patients with ECMO initiation 5 days after ARDS onset. Variable with statistical significance is marked with an asterisk (*).

Variable	Early ECMO (*n* = 12)	Delayed ECMO (*n* = 4)	*p*-Value
Age (years)	54.2 ± 16.2	60.5 ± 21.1	0.538
GCS score	10.5 ± 3.4	9.5 ± 3.4	0.601
Pre-ECMO PaO_2_/FiO_2_ ratio	68.0 ± 15.1	68.2 ± 16.1	0.977
ECMO duration (days)	11.3 ± 6.6	26.8 ± 12.1	0.014 *
ICU length of stay (days)	29.9 ± 28.5	49.3 ± 50.1	0.409
Hospital length of stay (days)	44.4 ± 28.5	81.0 ± 50.1	0.204
Hospital survival (%)	33.3%	75.0%	0.266
ECMO weaning (%)	58.3%	75.0%	0.619
mRS score at discharge	4.58 ± 2.15	3.50 ± 1.91	0.408

## Data Availability

Data available in a publicly accessible repository.
